# Beyond Amyloid: Systemic and Brain Frailty as Determinants of Response to Anti-Amyloid Therapy in Alzheimer’s Disease—A Conceptual Review

**DOI:** 10.3390/medicina62081489

**Published:** 2026-08-02

**Authors:** Polona Rus Prelog, Matija Zupan, Mišo Šabović, Senta Frol, Milica Gregorič Kramberger

**Affiliations:** 1 Centre for Clinical Psychiatry, University Psychiatric Clinic Ljubljana, 1260 Ljubljana, Slovenia; 2 Faculty of Medicine, University of Ljubljana, 1000 Ljubljana, Slovenia; matija.zupan@kclj.si (M.Z.); senta.frol@kclj.si (S.F.);; 3 Department of Vascular Neurology, University Medical Centre Ljubljana, 1000 Ljubljana, Slovenia; 4 Department of Vascular Diseases, University Medical Centre Ljubljana, 1000 Ljubljana, Slovenia; 5 Department of Neurology, University Medical Centre Ljubljana, 1000 Ljubljana, Slovenia; 6 Department of Neurobiology, Care Sciences and Society (NVS), Division of Clinical Geriatrics, Karolinska Institutet, 14157 Huddinge, Sweden

**Keywords:** Alzheimer’s disease, frailty, brain frailty, systemic frailty, monoclonal antibodies, amyloid-related imaging abnormalities (ARIA), precision medicine, cerebral small vessel disease, risk-benefit stratification

## Abstract

Anti-amyloid therapy (AAT) with monoclonal antibodies (mAbs) modestly slow cognitive and functional decline in early Alzheimer’s disease (AD). However, both the magnitude of clinical benefit and the risk of treatment-related complications vary substantially even among patients with similar biomarker profiles. Frailty, both brain and systemic, is highly prevalent in older adults with AD and affects a large proportion of those considered for AAT. Despite this, it has been largely absent from current decision frameworks. Brain frailty, defined by structural and microvascular damage (e.g., small-vessel disease, microbleeds, and atrophy), limits the clinical benefit of amyloid clearance and increases susceptibility to amyloid-related imaging abnormalities. In contrast, systemic frailty, reflecting reduced physiological reserve, mainly affects treatment tolerance and recovery from adverse events. In this narrative, conceptual review, we synthesize evidence that both forms of frailty act as biologically grounded modifiers of AAT efficacy and safety and may limit the clinical benefit while increasing susceptibility to complications and decompensation. Importantly, the precise empirical thresholds at which frailty begins to exert harmful effects remain unknown. We further outline how MRI-based markers of brain frailty, combined with brief systemic frailty measures, could support risk stratification, patient selection, monitoring intensity, and shared decision-making, including deferring treatment when the benefit–risk balance is unfavorable, while avoiding exclusion of patients who may still benefit. Taken together, we propose that future studies should incorporate frailty measures and perform precise assessments of both brain and systemic frailty, as this may improve patient stratification and better characterize the effects of AAT.

## 1. Introduction

Anti-amyloid therapy (AAT) with monoclonal antibodies (mAbs) has marked an important turning point in the treatment of Alzheimer’s disease (AD), demonstrating that targeted removal of amyloid can modestly slow cognitive and functional decline in appropriately selected patients in the early clinical stages [1]. At the same time, its use has revealed how narrow our current decision frameworks are: eligibility and monitoring are still dominated by biomarker status and genetics, while broader features of patient vulnerability and resilience receive comparatively little attention [1,2,3]. The key clinical question is not only whether amyloid can be removed, but under which conditions this removal is most likely to translate into preserved cognition, independence, and quality of life over time.

Systemic frailty, conceptualized as reduced multisystem physiological reserve and increased vulnerability to stressors, is common in older adults with cognitive impairment and dementia and is consistently linked with poorer functional outcomes and increased mortality [4]. In parallel, neuroimaging and clinical work suggests that many patients also exhibit brain frailty: an accumulation of structural and microvascular brain insults, including white matter hyperintensities (WMHs) (i.e., leukoaraiosis), lacunes, microbleeds, and cerebral atrophy, that reflect reduced brain reserve and resilience to further injury [5,6]. These systemic and cerebral forms of frailty are highly prevalent in “real-world” Alzheimer populations but are underrepresented in pivotal mAb trials due to exclusion of extensive vascular pathology and comorbidities [7]. As a result, we still lack a coherent framework for understanding how systemic and brain frailty might shape both the benefits and risks of AAT in real world populations.

In this article, we propose an integrated frailty-centered framework in which systemic frailty and brain frailty may act as biologically grounded modifiers of AAT response in AD. Rather than questioning the disease-modifying role of mAbs, we aim to explain why patients with similar amyloid and tau biomarker profiles can have very different clinical trajectories. We conceptualize frailty as a potential determinant of resilience: systemic frailty reflects overall multisystem vulnerability, and brain frailty reflects structural and vascular vulnerability within networks supporting cognition and daily functioning, together shaping both benefit and risk. The framework proposed here is hypothesis-generating and has not yet been prospectively validated for treatment selection or prediction of anti-amyloid therapy outcomes.

## 2. Methods

This article is a narrative, conceptual review rather than a systematic review or meta-analysis. Accordingly, we did not perform a formal risk-of-bias assessment of individual studies, and no quantitative data pooling was undertaken. Our aim was to integrate current evidence on anti-amyloid monoclonal antibodies (mAbs) in Alzheimer’s disease (AD) with emerging concepts of systemic and brain frailty, and to develop a frailty-centered framework for understanding and predicting treatment benefit and harm in real-world patient populations. To support transparency and breadth, we used a structured search strategy and thematic synthesis while retaining flexibility to incorporate mechanistic, clinical, and ethical perspectives.

### 2.1. Information Sources and Search Strategy

We conducted a structured literature search in PubMed/MEDLINE and Embase for articles published from 1 January 2000 to 31 March 2026. The search was designed to capture the literature relevant to anti-amyloid monoclonal antibodies in Alzheimer’s disease, systemic frailty, brain frailty, cerebral small-vessel disease, and treatment-related outcomes, and combined controlled vocabulary terms with free-text keywords adapted to each database syntax. Search terms were grouped into four concept blocks: (1) Alzheimer’s disease and related syndromes; (2) anti-amyloid monoclonal antibodies and amyloid-related imaging abnormalities; (3) systemic frailty and vulnerability; and (4) brain frailty and neuroimaging markers of cerebral small-vessel disease. The main terms included “Alzheimer’s disease,” “anti-amyloid,” “monoclonal antibody,” “aducanumab,” “lecanemab,” “donanemab,” “amyloid-related imaging abnormalities,” “ARIA,” “frailty,” “frailty index,” “frailty phenotype,” “brain frailty,” “cerebral small-vessel disease,” “white matter hyperintensities,” “microbleeds,” “cerebral amyloid angiopathy,” “atrophy,” and “neurovascular unit.”

We limited the search to peer-reviewed English-language publications and supplemented database searching by screening the reference lists of pivotal anti-amyloid trial reports, recent reviews, consensus statements, and guideline papers relevant to treatment selection, ARIA risk, frailty, and cerebral small-vessel disease. We preferentially included studies and reviews that directly informed the conceptual framework, including pivotal trials of lecanemab and donanemab, observational studies on frailty and MRI markers of brain frailty, and recent reviews and consensus papers on anti-amyloid therapy and patient selection. Titles and abstracts were screened first, followed by full-text review of potentially relevant articles. Because this was a narrative conceptual review, we did not apply formal PRISMA selection procedures, risk-of-bias scoring, or quantitative meta-analysis.

### 2.2. Inclusion and Exclusion Criteria

We included peer-reviewed articles in English that met at least one of the following criteria: (i) pivotal phase II–III trials or large observational studies of anti-amyloid mAbs in early symptomatic AD; (ii) studies examining systemic frailty using validated measures (e.g., frailty index, frailty phenotype) in older adults with cognitive impairment, dementia, or closely related populations; (iii) studies investigating imaging markers of brain frailty and cerebral small-vessel disease (e.g., white matter hyperintensities, lacunes, microbleeds, cortical or subcortical atrophy) in relation to cognitive, functional, or mortality outcomes; or (iv) authoritative guidelines, consensus statements, or conceptual papers on anti-amyloid therapy, frailty, or ethical decision-making in dementia care. We excluded isolated case reports and very small case series (typically fewer than 10 participants) without clear mechanistic relevance, editorials and commentaries without primary data or substantive conceptual contribution, conference abstracts without full data, and non-English publications without an accessible translation.

### 2.3. Synthesis

Eligible sources were appraised for their conceptual and clinical relevance to our central question: how do systemic and brain frailty modify the benefits and risks of anti-amyloid mAbs in AD, and how can these constructs be operationalized to guide real-world treatment decisions? As this is a narrative review, we did not apply formal scoring tools or conduct meta-analyses; quantitative findings from individual studies and previously published meta-analyses are cited as originally reported. Evidence is organised thematically into: (i) definitions and operationalisation of systemic and brain frailty; (ii) their prevalence and prognostic significance in AD; (iii) current evidence on the efficacy and safety of anti-amyloid mAbs; (iv) mechanistic links between frailty, neurovascular vulnerability, and limited treatment effectiveness; and (v) implications for patient selection, monitoring, ethical decision-making, and future research priorities.

## 3. Frailty Concepts

Frailty refers to a state of multisystem physiological vulnerability characterized by diminished capacity to withstand external stressors [8] and is associated with increased morbidity and mortality across a wide range of medical conditions [9], including neurodegenerative disease. While frailty commonly coexists with advanced age, multimorbidity, and disability [10], it represents a distinct clinical syndrome: prevalence increases sharply with age [11], yet considerable heterogeneity in biological aging means that chronological age alone does not capture frailty-related risk [10].

Two dominant conceptual frameworks are used to assess frailty: the cumulative deficit model (i.e., the Rockwood model) [12] and the frailty phenotype model (i.e., the Fried model) [13]. The cumulative deficit model (“Rockwood”) quantifies frailty by tallying equally weighted deficits across multiple domains, such as cerebrovascular disease, atrial fibrillation, conventional vascular risk factors, incontinence, cognitive impairment, cancer, and certain laboratory abnormalities [12], whereas frailty phenotype model (“Fried”) defines frailty based on five physical characteristics: unintentional weight loss, exhaustion, low activity, slow gait speed, and reduced grip strength [12,13].

In parallel, the construct of brain frailty has emerged. Brain frailty represents reduced neurophysiological reserve and increases vulnerability to poor cognitive outcomes [14]. It is defined by routinely identifiable neuroimaging markers on baseline CT or MRI, including cortical and subcortical atrophy, leukoaraiosis, chronic infarcts, lacunes, enlarged perivascular spaces, and microhemorrhages [15]. These radiologic features largely reflect cerebral small-vessel disease (CSVD) [15].

Existing approaches include a three-item brain-frailty score based on the presence of leukoaraiosis, cerebral atrophy, and old vascular lesions [5], and a total cerebral small vessel disease (CSVD) score ranging from 0 to 4, which integrates white-matter hyperintensities (WMHs), lacunes, cerebral microbleeds, and enlarged perivascular spaces [16]. Although these measures have demonstrated prognostic value in stroke and ageing cohorts, neither has been validated as an AD-specific treatment-selection instrument, and the extent to which imaging-based brain frailty overlaps with systemic or physical frailty remains incompletely understood. We provide a comparison between different frailty models in Table 1.

Recent conceptual work suggests that systemic frailty and brain frailty are related but only partially overlapping constructs: systemic frailty captures overall multisystem physiological reserve, whereas brain frailty captures structural and microvascular brain vulnerability that may mediate how systemic vulnerability manifests cognitively. Both are prevalent in real-world patients with AD, yet frailty—particularly brain frailty—has received limited, mostly indirect attention in pivotal AAT mAbs trials, which have tended to exclude patients with significant vascular pathology and other markers of high frailty [1,17,18].

For clarity, these related constructs should not be treated as interchangeable. Disease burden denotes the amount of established pathology, including tau-mediated neurodegeneration and cerebral small vessel disease (CSVD). Brain reserve refers to the structural capacity available to tolerate such pathology, whereas cognitive reserve refers to the functional adaptability that permits cognition to be maintained despite it. Brain frailty describes the integrated vulnerability arising from accumulated structural and vascular injury and a diminished ability to withstand additional stressors. A single marker may therefore have different interpretive roles: atrophy is principally a manifestation of neurodegeneration, but extensive atrophy also indicates reduced brain reserve and may contribute to a composite brain-frailty assessment. Similarly, CSVD represents vascular disease burden, while its cumulative consequences, including white-matter hyperintensities, lacunes, microbleeds, and network disruption, contribute to brain frailty. Tau burden should therefore be regarded as a marker of AD-related neurodegeneration rather than as a frailty measure itself.

## 4. Interaction Between Systemic and Brain Frailty

Systemic frailty and brain frailty appear to be bidirectionally linked, sharing upstream drivers such as vascular risk factors, chronic low-grade inflammation, and metabolic dysregulation. Population-based MRI studies show that frail and pre-frail older adults have a higher burden and more complex morphology of WMHs and other cerebral small-vessel disease markers than non-frail peers, suggesting that systemic vulnerability and vascular brain injury frequently co-occur [19]. In the context of anti-amyloid therapy, milder forms of brain frailty, such as diffuse white-matter changes, atrophy, and subtle microangiopathy, may reflect vulnerability that contributes to cognitive and functional impairment, whereas more advanced CSVD may indicate a higher-vulnerability phenotype requiring greater caution [19,20,21,22]. Taken together, these observations support the hypothesis that brain and systemic frailty are overlapping but distinct dimensions of vulnerability that may influence the balance between potential benefit and risk, although this remains to be prospectively validated.

## 5. Brain Frailty and Alzheimer’s Disease

In Alzheimer’s disease, brain frailty offers one plausible explanation for why patients with a similar burden of amyloid pathology can show markedly different symptom severity and rates of decline, as imaging-based markers of CSVD and atrophy consistently predict steeper cognitive and functional deterioration. While tau accumulation is a key marker of neurodegeneration and aligns more closely with clinical stage than amyloid alone, post hoc analyses of donanemab trials (TRAILBLAZER-ALZ and TRAILBLAZER-ALZ 2) suggest that participants with lower or intermediate baseline tau burden experience numerically greater slowing of clinical decline than those with high tau burden, supporting the concept of a therapeutic window in which brain reserve is still partly preserved [23,24]. Within this window, lower brain frailty—reflected by less CSVD and atrophy—likely contributes to greater cognitive resilience, whereas high brain frailty, characterized by extensive white matter damage, microbleeds, and atrophy, is associated with earlier and steeper clinical decline even at comparable amyloid levels [25]. Conceptually, brain frailty overlaps with but remains distinct from brain and cognitive reserve. Disease burden describes the pathology present, reserve describes the capacity to tolerate or compensate for it, and brain frailty describes the resulting vulnerability to additional stress, functional decline, and loss of independence [5,26].

## 6. Systemic Frailty and Alzheimer’s Disease

Systemic frailty, like brain frailty, appears to contribute to the clinical heterogeneity of AD, including why individuals with similar amyloid burden can differ markedly in symptom severity and trajectories of decline [27]. Prospective cohort studies show that higher frailty is associated with increased risk of incident dementia, faster cognitive decline, and earlier loss of independence, even after accounting for age, comorbidity, and imaging markers of brain atrophy and CSVD, suggesting that multisystem vulnerability modifies how underlying pathology translates into clinical expression.

Low systemic frailty reflects greater physiological resilience, in which the brain and body are better able to compensate for amyloid and tau pathology, whereas high systemic frailty is linked to steeper functional decline and higher rates of ADL and IADL dependency, particularly when it coexists with cognitive impairment.

## 7. Monoclonal Antibody Therapy in Alzheimer’s Disease: Current Evidence

AAT mAbs bind aggregated amyloid-β and promote its clearance via microglial phagocytosis and downstream proteolytic and vascular efflux pathways, leading to substantial reductions in amyloid PET signal and plaque burden in early symptomatic AD. Their effects depend on relatively intact neuroimmune and vascular systems: microglial activation, blood–brain barrier function, and perivascular drainage must be sufficient to remove antibody–amyloid complexes without causing excessive edema or hemorrhage [1].

Clinically, they produce modest slowing of cognitive and global decline, typically on the order of 20–30% over 18–24 months in carefully selected, biomarker-positive early AD cohorts, while effects on everyday functioning and quality of life remain limited [28,29,30]. Treatment is accompanied by a characteristic safety profile dominated by amyloid-related imaging abnormalities, with vasogenic edema (ARIA-E) and microhemorrhages or superficial siderosis (ARIA-H) occurring in a substantial minority of treated patients, particularly among APOE ε4 carriers [31,32,33], necessitating regular MRI surveillance, possible dose interruptions or discontinuation, and careful discussion of monitoring burden and risk–benefit with patients and caregivers.

The recent Cochrane meta-analysis of anti-amyloid therapies for Alzheimer’s disease [34] provided a rigorous data synthesis, but it was largely driven by compounds that failed regulatory approval, with only a minority of studies evaluating clinically relevant agents like lecanemab and donanemab. The analysis risked conflating treatments supported by markedly different levels of evidence and risked obscuring the specific benefits and risks of currently available treatments [35]. Full clinical significance remains under further evaluation through ongoing long-term extension studies and real-world evidence, including frailty-based and functional outcomes that may better capture meaningful benefits in older adults.

## 8. Mechanistic Links Between Systemic Frailty, Brain Frailty, and Treatment Outcomes

Systemic and brain frailty offer a biologically plausible rationale for why robust amyloid clearance may not translate into proportional, durable clinical benefit. Brain frailty is associated with greater structural and vascular disease burden—including cerebral and hippocampal atrophy and greater WMH burden—which reduces brain reserve and limits the capacity of cognitive reserve to compensate once neurodegeneration and CSVD are established [36]. In this context, amyloid removal can stabilize one component of disease biology without restoring the structural and functional substrate required for meaningful recovery of cognition and independence, particularly when tau-mediated neurodegeneration and other non-amyloid processes have become dominant drivers of decline [37].

**Figure 1:**
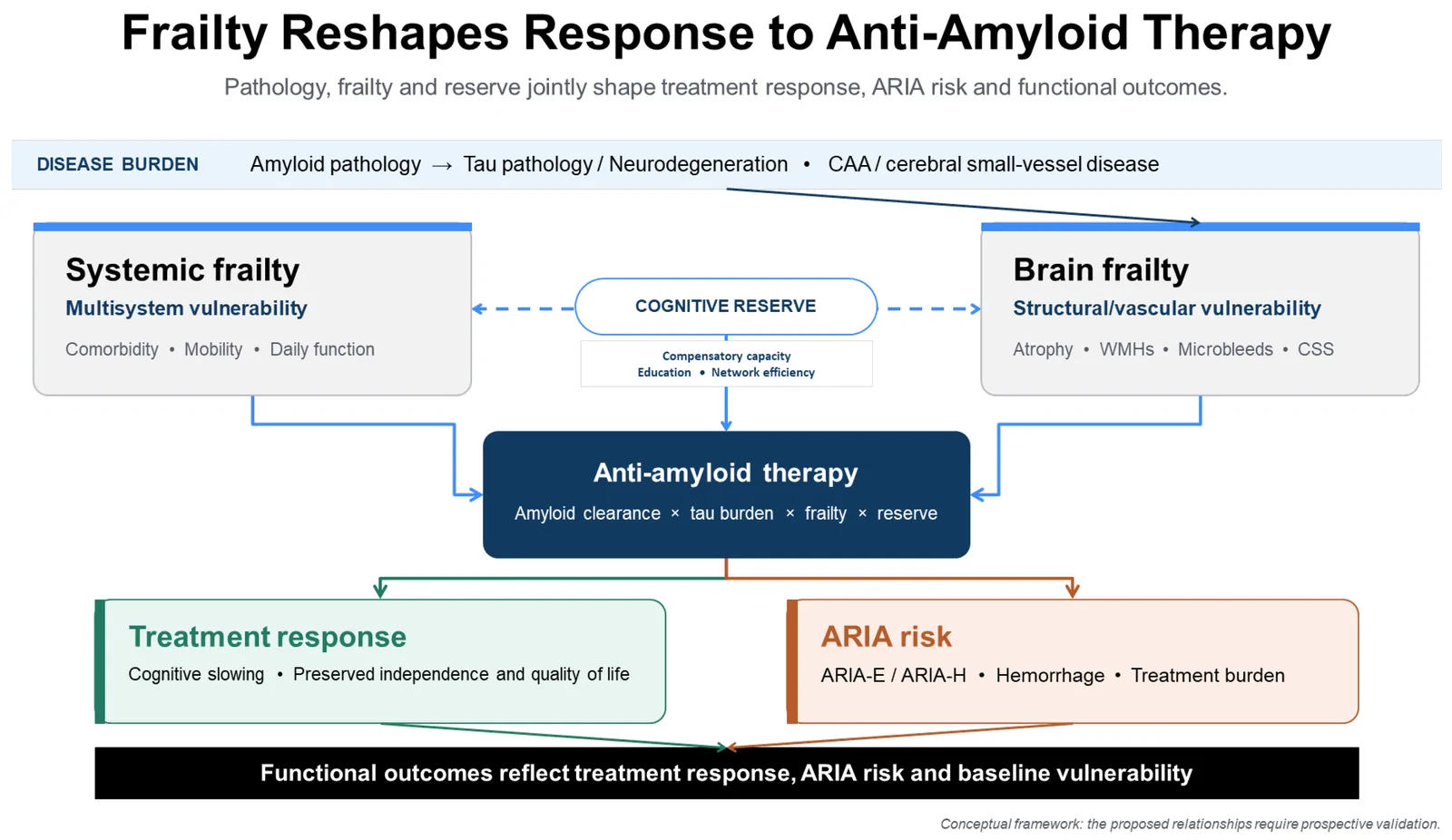
Proposed framework for how systemic and brain frailty modify the benefit–risk balance of anti-amyloid therapy. ARIA: amyloid-related imaging abnormalities; ARIA-E: amyloid-related imaging abnormalities-edema; ARIA-H: amyloid-related imaging abnormalities-hemorrhage; CAA: cerebral amyloid angiopathy; CSS: cortical superficial siderosis; WMHs: white matter hyperintensities.

Systemic frailty contributes additional constraints through “inflammaging,” microglial dysregulation, and multisystem metabolic and immune impairment [38,39,40], which can dampen antibody-mediated amyloid clearance and heighten vulnerability to complications. Conceptually, these interacting pathways may create a functional ceiling: higher systemic and brain frailty bound the extent to which biomarker changes can be converted into sustained gains in cognition, activities of daily living, and quality of life [17,41], even when mAbs achieve their proximal pharmacodynamic targets. Figure 1 summarizes our proposed conceptual framework, in which systemic and brain frailty interact to modify the extent to which amyloid clearance translates into meaningful clinical benefit while simultaneously influencing the risk of treatment-related harm.

### Cerebral Amyloid Angiopathy, APOE ε4, and ARIA Mechanisms

Cerebral amyloid angiopathy (CAA), characterized by amyloid-β deposition within cortical and leptomeningeal vessel walls [42], provides an important mechanistic link between brain frailty and AAT-related harm. CAA reflects—and may further impede—perivascular amyloid clearance while weakening vascular integrity and the neurovascular unit [43]. Human neuropathological studies following amyloid-β immunization demonstrated that plaque removal can be accompanied by transient redistribution of amyloid-β and APOE towards vessel walls, increased vascular amyloid burden, vessel-wall alterations, and microhemorrhages [43]. APOE ε4 further favors vascular amyloid accumulation [3] and is associated with a dose-dependent increase in ARIA risk [44]. Antibody-mediated amyloid mobilization may consequently overload already fragile vessels, with increased vascular permeability and perivascular inflammation producing vasogenic edema or sulcal effusion (ARIA-E), whereas leakage or rupture of amyloid-laden vessels produces microhemorrhages and cortical superficial siderosis (ARIA-H) [43,44]. Although the precise sequence remains incompletely understood, CAA is therefore a biologically plausible component of brain frailty that may amplify treatment-related harm despite successful amyloid clearance.

## 9. Brain and Systemic Frailty and the Benefit–Risk Balance of Monoclonal Antibody Therapy

In clinical decision-making, the key question is not only whether AAT removes amyloid, but also how far this removal can be expected to translate into benefit relative to risk in individual patients. In our framework, brain and systemic frailty jointly shape this benefit–risk balance by influencing both the likelihood of meaningful clinical response and the susceptibility to treatment-related harm.

### 9.1. Brain Frailty as a Predictor of Efficacy

Higher baseline brain frailty may attenuate treatment effectiveness because reduced brain and cognitive reserve limit compensatory network reorganization once amyloid is removed [5]. In contrast, patients with lower brain frailty may be better able to translate plaque clearance into slower cognitive decline and preserved daily functioning, particularly when tau burden and overall neurodegeneration remain in an earlier, more treatment-responsive stage, as suggested by subgroup analyses of donanemab and other second-generation antibodies in low/intermediate tau strata [24].

### 9.2. Brain Frailty as a Predictor of Harm

Brain frailty also signals a vulnerable neurovascular substrate. Chronic microvascular damage and coexisting CAA create fragile, amyloid-laden vessels in which rapid perivascular amyloid clearance can precipitate vasogenic edema (ARIA-E) and microhemorrhages or superficial siderosis (ARIA-H). In this context, higher brain frailty and heavier CSVD burden, particularly in APOE ε4 carriers, who have substantially increased ARIA incidence and severity in pooled analyses [33,45,46] may be associated with more frequent or severe ARIA, slower radiological and clinical recovery, and a greater likelihood of dose interruption or early discontinuation, although these hypotheses still need formal testing in frailty-stratified datasets.

### 9.3. Systemic Frailty as a Predictor of Efficacy

Systemic frailty may provide a second layer of constraint on therapeutic benefit. Frail patients typically have multimorbidity (e.g., cardiovascular disease, diabetes, renal impairment), impaired metabolic and immune responses, and polypharmacy [38], all of which can reduce the efficiency of AAT and narrow the physiological margin for improvement. By analogy with other biologic and immunotherapies in older adults [47], frailty is associated with shorter survival, greater treatment burden, and higher treatment discontinuation, suggesting that systemic frailty may similarly constrain the net benefit of anti-amyloid therapy; however, this remains an indirect inference rather than direct evidence.

### 9.4. Systemic Frailty as a Predictor of Harm

As a predictor of harm, systemic frailty captures vulnerability to both neurological and systemic complications of treatment. Frail individuals are more prone to infections, delirium, falls, decompensation of chronic disease, and prolonged hospitalization following adverse events [48,49], so ARIA or other adverse effects that might be transient in robust patients can trigger sustained functional decline and loss of independence. In addition, interactions between systemic frailty, APOE genotype, vascular comorbidities, and concomitant medications such as antithrombotic agents can further increase the risk that treatment-related events lead not only to radiological abnormalities but to clinically meaningful harm [4,17,50], reinforcing the need to integrate frailty assessment into benefit–risk discussions for AAT.

## 10. Integrating Brain and Systemic Frailty into Clinical Selection

Incorporating assessments of brain and systemic frailty may improve patient selection for therapy, allowing for a more individualized approach and better prediction of both efficacy and risk.

### Practical MRI Operationalization of Brain Frailty

At present, no MRI-derived brain-frailty score has been prospectively validated specifically for AD treatment selection. Existing measures nevertheless provide a pragmatic starting point: the three-item brain-frailty score assigns one point each for leukoaraiosis, cerebral atrophy, and old vascular lesions, whereas the conventional total CSVD score assigns one point each for severe WMHs—periventricular Fazekas grade 3 and/or deep Fazekas grades 2–3—at least one lacune, at least one cerebral microbleed, and moderate-to-severe basal-ganglia perivascular spaces, producing a total score of 0–4 [5,16,51]. In an AD clinic, these measures could be extended into a structured MRI profile based on T1-weighted, FLAIR, and susceptibility-sensitive T2*-GRE or SWI sequences, with lesions defined according to STRIVE/STRIVE-2 criteria [15,52]. Minimum reporting should include global cortical and medial temporal atrophy; WMH severity using the Fazekas scale and, where validated software is available, WMH volume normalized to intracranial volume; lacune number and location; and microbleed number and distribution—strictly lobar versus deep or infratentorial—using MARS or an equivalent validated scale [51,53]. Cortical superficial siderosis should be recorded separately as absent, focal (≤3 sulci), or disseminated (≥4 sulci), because it may indicate advanced CAA and greater hemorrhagic vulnerability [54]. Importantly, both the composite score and its individual components should be reported, because identical total scores may conceal markedly different CAA and ARIA risk; baseline microhemorrhages, lobar microbleeds, and cortical superficial siderosis have been associated with an increased likelihood and potential severity of ARIA during AAT [55,56,57,58]. Until prospectively validated in anti-amyloid cohorts, this structured profile should support—not replace—approved eligibility criteria and multidisciplinary clinical judgement.

Including systemic frailty alongside MRI data provides a more complete picture of patient vulnerability; patients with significant cardiovascular comorbidity, poor functional reserve, or polypharmacy are more prone to systemic complications and decompensation in response to treatment-related events [17], which can compromise tolerance and recovery. Combining brain and systemic frailty within an MRI-based stratification framework may allow simultaneous assessment of neurological and multisystem risk, enabling a personalized risk profile for each patient and more informed shared decision-making about whether and how to proceed with AAT.

## 11. Implications for Clinical Practice

Combined assessment of brain and systemic frailty may support more individualized monitoring and dose-adjustment strategies in patients receiving AAT. Patients with high brain frailty may warrant more frequent MRI surveillance, slower titration, and closer neurological follow-up, whereas patients with high systemic frailty require careful monitoring for systemic complications, delirium, falls, and functional decline, consistent with frailty guidelines [49,59]. This integrated, frailty-informed approach may help optimize therapy by safely including some patients who might otherwise be excluded under rigid trial-derived criteria, while simultaneously identifying those in whom the cumulative risk of ARIA, hospitalization, or loss of independence is likely to outweigh the modest expected benefit of treatment.

## 12. Ethical and Clinical Decision-Making

AAT mAbs force clinicians to navigate a tension between “treating amyloid in a frail brain” and treating the whole patient. In frail older adults, independence, avoidance of hospitalization, and quality of life may matter more than modest changes in cognitive scores or amyloid PET, and that interventions which increase falls, ARIA, or caregiver burden can undermine these outcomes [60]. Frailty, comorbidity, and functional status should be treated as central elements of beneficence and non-maleficence, not as peripheral modifiers once eligibility has already been assumed.

Communicating probabilistic benefit becomes particularly challenging when brain frailty limits the translation of amyloid clearance into durable clinical gains and when long-term effects remain uncertain. Ethical guidance on AAT immunotherapy recommends explicitly discussing the modest magnitude of benefit, the possibility of no noticeable improvement, and the potential for treatment to worsen autonomy or independence through ARIA or treatment burden [61,62]. Decision aids and structured supported decision-making can help patients and care partners engage with these uncertainties, while recognising that many rely heavily on family members to interpret and act on probabilistic information.

In this setting, shared decision-making should integrate imaging findings (amyloid, tau, and brain frailty markers), systemic frailty, and current functional status, and should make explicit that “not treating” can be an ethically legitimate choice when the expected balance of benefit and harm is unfavorable. Clinicians are encouraged to frame AAT as an option added onto a foundation of high-quality supportive care [62], rather than as a default or obligatory step, and to revisit decisions over time as frailty, comorbidity, and patient priorities evolve.

## 13. Future Perspectives

Future work should focus on integrating systemic and brain frailty into combined indices that capture overall resilience and vulnerability in a clinically usable way. Such indices should provide the foundation for prospectively validated, composite frailty-risk algorithms that estimate treatment response and adverse events and help clinicians and patients weigh expected benefit against potential harm more systematically.

Prospective, frailty-stratified registries should enroll consecutive patients across the full spectrum of systemic and brain frailty, apply harmonized baseline assessments, and capture not only ARIA and treatment discontinuation but also cognitive and functional trajectories, falls, hospitalisation, institutionalisation, caregiver burden, and quality of life. In parallel, future AAT trials should incorporate validated systemic frailty instruments and standardized MRI-based brain frailty measures at baseline, avoid unjustified exclusion of frail individuals, and prespecify frailty subgroup and treatment-by-frailty interaction analyses. Such analyses should distinguish whether frailty is merely prognostic or genuinely modifies treatment effects across efficacy, ARIA, serious adverse events, treatment burden, and patient-centered outcomes.

A frailty-centered perspective also supports testing therapeutic combinations that aim to enhance brain resilience as well as remove amyloid, for example pairing AAT with intensive vascular risk factor modification, optimized treatment of CSVD, and selected neuroprotective or neurorestorative interventions. Outcome assessment should move beyond amyloid clearance and short-term cognitive scores to include trajectories of brain frailty and functional preservation—such as activities of daily living, independence, and quality of life—as key endpoints.

The resulting datasets could support composite frailty-risk algorithms that integrate systemic frailty with age, APOE ε4 status, comorbidity, functional status, treatment exposure, and MRI markers, including white-matter hyperintensities, lacunes, cerebral microbleeds, cortical superficial siderosis, and atrophy. Machine-learning models may identify nonlinear interactions among these variables and generate individualized estimates of net clinical benefit; however, they should be developed in representative multicenter cohorts, externally and prospectively validated, assessed for calibration, discrimination, clinical utility, and fairness, and benchmarked against simpler transparent scores before clinical implementation.

Translating these concepts into practice will require simple ways to measure combined frailty at the bedside. A pragmatic approach could couple a brief systemic frailty measure with a standardized MRI-based brain frailty score, summarized into a small number of risk strata (for example low, intermediate, and high frailty), and embed into routine pathways from primary care triage through memory clinic evaluation to pre-treatment work-up.

## 14. Conclusions

Systemic and brain frailty together may help explain some of the heterogeneity in treatment response and adverse event risk seen with AAT mAbs. In our framework, AAT may be most clinically effective—and safest—in patients with both low systemic frailty and low brain frailty, where sufficient physiological and structural reserve remains to convert amyloid removal into preserved cognition, independence, and quality of life.

Future Alzheimer’s therapeutics should therefore integrate markers of systemic and brain resilience alongside amyloid and tau biomarkers, with implications for both clinical practice and trial design. Assessing the whole patient—capturing multisystem physiological reserve and structural brain vulnerability—can support more realistic discussions of benefit and risk, more appropriate patient selection, and more meaningful outcome measures.

## Figures and Tables

**Table 1:** Comparison of different frailty models.

Feature	Rockwood Frailty Index (Cumulative Deficit Model)	Fried Frailty (Phenotype Model)	Brain Frailty
**Core Concept**	Frailty as the accumulation of health deficits	Frailty as a physical phenotype reflecting reduced physiological reserve	Frailty as reduced neurophysiological and structural brain reserve
**Primary Domains Assessed**	Multisystem deficits (comorbidities, mobility, cognition, labs, symptoms, disabilities)	Physical performance and muscle function	Neuroimaging markers (structural brain integrity and chronic injury)
**Assessment Method**	Counting the proportion of deficits present out of a predefined list (typically 30–70 items)	Presence of ≥3 of 5 physical criteria (weight loss, exhaustion, low activity, slow gait, weak grip)	Visual rating of CT/MRI markers of chronic brain pathology
**Approximate assessment time**	Approximately 10–20 min when the required clinical data are available; substantially shorter when calculated automatically from electronic health records	Approximately 5–10 min, including gait-speed and grip-strength testing	Approximately 5–10 min for visual scoring once suitable CT/MRI has been acquired; imaging acquisition time is additional
**Typical clinical setting**	Comprehensive geriatric assessment, memory clinics, inpatient or outpatient geriatric care, research registries, and electronic-health-record screening	Primary care, geriatric and rehabilitation clinics, community screening, and clinical-trial assessments	Memory and stroke clinics, neuroradiology assessment, and pretreatment imaging evaluation for AAT
**Type of Measurement**	Continuous index (0 → 1)	Categorical (robust, pre-frail, frail)	Ordinal/categorical (depending on scoring system used)
**Key Inputs**	Medical history, comorbidities, ADLs/IADLs, cognitive tests, blood tests	Gait speed, grip strength, questionnaires, weight measurements	CT/MRI: atrophy, leukoaraiosis, lacunes, chronic infarcts, microbleeds, enlarged perivascular spaces
**Examples of Variables**	Prior stroke, AF, cancer, incontinence, cognitive impairment, anemia	Slow walking speed, weak grip strength, low physical activity	White matter hyperintensity burden, cortical atrophy, lacunes
**Interpretation**	Higher index → more deficits → higher frailty	Greater number of physical phenotypic criteria → higher frailty	More neuroimaging abnormalities → greater brain frailty
**Validation evidence**	Extensively validated across community and hospital populations for mortality, disability, hospitalization, institutionalization, and healthcare use; direct validation for predicting AAT outcomes remains limited	Extensively validated in community-dwelling and clinical older populations for falls, disability, hospitalization, and mortality; AAT-specific validation is lacking	Observationally validated in ageing and cerebrovascular populations for cognitive decline, functional outcome, and mortality; definitions remain heterogeneous, and prospective validation for AAT outcomes or ARIA is lacking
**Potential for predicting treatment benefit**	May identify patients with sufficient multisystem reserve to realise modest cognitive and functional benefits, but has not been established as a predictor of differential AAT efficacy	Physical robustness may support treatment adherence and preservation of independence, but the phenotype has not been shown to predict cognitive response to AAT	Lower structural and vascular brain burden may indicate greater capacity to translate amyloid clearance into clinical benefit; however, treatment-by-brain-frailty interactions remain unproven
**Potential for predicting treatment-related harm**	Particularly informative for general vulnerability to hospitalization, delirium, falls, functional decline, and treatment burden, but relatively nonspecific for ARIA	Useful for identifying susceptibility to falls, deconditioning, and loss of independence; less informative for imaging-defined or neurological complications	Potentially the most directly relevant model for ARIA, ICH, and reduced neurological resilience because it incorporates microbleeds, CSS, WMHs, and atrophy; nevertheless, a composite brain-frailty score has not been prospectively validated for AAT safety
**Practical Strengths**	Holistic assessment of multisystem vulnerability; predictive of mortality and functional decline; can use routinely collected clinical data and be automated	Brief, inexpensive, reproducible, and readily interpretable; directly measures physical performance	Uses clinically indicated imaging, adds little patient burden when MRI is already planned, and captures brain-specific structural and vascular vulnerability and biological aging
**Limitations**	Time-consuming; requires many variables; heterogeneous datasets	Narrow (physical only); may miss cognitive or systemic components	Lacks standardized consensus definition; less studied; imaging quality and visual scoring varies
**Relationship With Aging**	Closely tied to biological (not chronological) aging	Captures physical components of aging	Reflects cerebral aging and small-vessel disease burden
**Overlap With Others**	Moderate overlap with Fried and brain frailty but assesses broader systemic factors	Partial overlap; correlates with physical components of Rockwood	Overlaps minimally; complementary to physical frailty measures

ADLs: activities of daily living: AF: atrial fibrillation; ARIA: amyloid-related imaging abnormalities; CSS: cortical superficial siderosis; CT: computerized tomography; IADLs: instrumental activities of daily living; ICH: intracranial hemorrhage; MRI: magnetic resonance imaging; WMHs: white matter hyperintensities. Assessment times are approximate and refer to scoring when the necessary clinical data or imaging are already available; they vary according to local workflow and degree of automation. Although all three models provide prognostic information, none has yet been prospectively validated as a treatment-selection tool or as a predictor of differential benefit from AAT.

## Data Availability

No new data were created or analyzed in this study. Data sharing is not applicable to this article.

## Abbreviations

The following abbreviations are used in this manuscript:

AAT	Anti-amyloid therapy
mAbs	Monoclonal antibodies
AD	Alzheimer’s disease
WMHs	White matter hyperintensities
CSVD	Cerebral small-vessel disease
CT	Computed tomography
MRI	Magnetic resonance imaging
PET	Positron emission tomography
ARIA	Amyloid-related imaging abnormalities
ARIA-E	Amyloid-related imaging abnormalities with edema/effusion
ARIA-H	Amyloid-related imaging abnormalities, hemorrhage (microhemorrhages/superficial siderosis)
APOE	Apolipoprotein E
APOE ε4	Apolipoprotein E epsilon-4 allele
ADLs	Activities of daily living
IADLs	Instrumental activities of daily living
AF	Atrial fibrillation
